# Characteristics of patients co-infected with HIV at the time of inpatient tuberculosis treatment initiation in Yaoundé, Cameroon: a tertiary care hospital-based cross-sectional study

**DOI:** 10.1186/s13690-015-0075-y

**Published:** 2015-05-04

**Authors:** Ako A Agbor, Jean Joel R Bigna, Claudia S Plottel, Serges Clotaire Billong, Mathurin Cyrille Tejiokem, Gabriel L Ekali, Jean Jacques N Noubiap, Roselyne Toby, Hermine Abessolo, Sinata Koulla-Shiro

**Affiliations:** Faculty of Medicine and Biomedical Sciences, University of Yaoundé 1, Yaoundé, Cameroon; Goulfey Health District, Goulfey, Cameroon; Department of Medicine, New York University Langone Medical Center, New York, USA; Department of Medicine, New York University School of Medicine, New York, USA; National AIDS Control Committee, Ministry of Public Health, Yaoundé, Cameroon; Department of Epidemiology and Public Health, Centre Pasteur du Cameroun, Member of International Network of the Pasteur Institutes, Garoua, Cameroon; Internal Medicine Unit, Edéa Regional Hospital, Edéa, Cameroon; Infectious Diseases Unit, Yaoundé Central Hospital, Yaoundé, Cameroon; Medical Diagnostic Center, Yaounde, Cameroon

**Keywords:** Tuberculosis, AIDS, HIV, Cameroon, Profile, TB & HIV co-infection, Resource-limited setting, Co-morbidities, TB

## Abstract

**Background:**

Knowledge of the characteristics of patients co-infected with tuberculosis (TB) and human immunodeficiency virus (HIV) when TB treatment is initiated would allow clinicians to improve care and help policy-makers develop relevant and realistic guidelines. The aim of this study was to describe socio-demographic, clinical, and laboratory characteristics of TB/HIV co-infected patients starting inpatient TB treatment in Yaoundé, Cameroon.

**Methods:**

We conducted a retrospective cross-sectional study, collecting data from medical records of HIV-infected patients with TB, aged 15 years old or more, hospitalized in the Infectious Diseases Unit of the Yaoundé Central Hospital, Cameroon from January 1, 2006 to June 30, 2013.

**Results:**

The mean age of 337 patients meeting study inclusion criteria was 39.3 years. More than half were female (53.4%). Most (89.3%) resided in urban areas, 44.2% had a secondary education, and 46.0% were married. The majority was receiving co-trimoxazole prophylaxis (79.5%), and two thirds were taking antiretroviral therapy (67.4%). The mean duration of known HIV infection before TB treatment was 8.4 months. Most (88.1%) had newly diagnosed TB, rather than relapsed disease. Smear-positive pulmonary TB was documented in a third, (35.3%). Laboratory data revealed a median white blood cell count of 5,100 cells/mm^3^ (IQR 3,300-7,990 cells/mm^3^), a median hemoglobin level of 8 g/dl (IQR 7–10 g/dl), and a median CD4 cell count of 102 cells/mm^3^ (IQR 33–178 cells/mm^3^). Sex differences in our study included older age in the men (*p* < 0.001), more of whom were married (*p* < 0.001) and had achieved a higher level of education (*p* = 0.042). Men had fewer diagnoses of smear-positive pulmonary TB (*p* = 0.020). They weighed more than the women (*p* = 0.001) and had higher hemoglobin levels (*p* = 0.003).

**Conclusions:**

Suboptimal adherence to WHO treatment recommendations in our Cameroonian study reinforces the importance of prescribing co-trimoxazole in HIV infection and ART for all TB/HIV co-infected persons. We urge that Ministries of Health continue implementing and disseminating guidelines for management of TB/HIV co-infected patients, and we call for measures ensuring that healthcare facilities’ stocks of ART and co-trimoxazole are sufficient to meet the need for both.

## Background

The Word Health Organization (WHO) estimates that the prevalence of tuberculosis (TB) in 2010 reached 12 million cases, corresponding to rates of 169 per 100,000 population worldwide and to 303 per 100,000 in Africa [1]. The Centers for Disease Control (CDC) point out that worldwide, approximately one third of persons are infected with *Mycobacterium tuberculosis* [2] underscoring the enormity of the global burden of TB. In most persons, TB infection remains latent and clinically quiescent; however in others, TB progresses and leads to active, infectious TB illness. The millennium development goals for global TB control are to halt and then, to begin reversing the increasing incidence of TB in order to halve the 1990 prevalence and death rates by 2015 [3]. Cameroon most recently recorded a total of 26,110 cases of TB nationally, with 15,080 new smear-positive cases, corresponding to rates of 124 and 73 per 100,000 population respectively [4]. The rate of successful tuberculosis treatment has been progressively increasing in Cameroon, from 72% in 2003 to 80% in 2013 [4].

People living with human immunodeficiency virus (HIV) are much more likely to develop active TB than those who are HIV-negative [1]. The greatest burden of combined TB and HIV infections is seen in sub-Saharan Africa, where the extent of the problem is tragically, inversely proportional to the paucity of resources available for effective control and treatment. Although sub-Saharan Africa has borne the brunt of TB/HIV co-infection and its consequences to date, other parts of the world are increasingly struggling with increasing TB and HIV-related morbidity and mortality as well. HIV infection is frequent among patients with active TB in Cameroon and disproportionately affects women [5,6]. Studies in Cameroon indicate that the HIV infection rate among TB patients has been rapidly increasing, from 16.6% in 1997 [7] to 51.6% in 2012 [8]. Recent estimates from CDC taking into account the fact that TB detection and reporting data from sub-Saharan Africa are often incomplete (underestimates), project that there will be 19,000 new HIV-positive TB patients in Cameroon alone in 2013 [2]. Reports from the Cameroonian Ministry of Public Health reveal that in 2013, 82% of patients known to have TB had also been tested for HIV; of those tested, 38% were HIV-infected; and of those co-infected with TB and HIV, 65% were being treated concurrently for both TB and HIV [4].

Despite the encouraging mortality figures from TB occurring in the setting of HIV, better knowledge and understanding of the presentation of TB/HIV co-infected patients at the time of TB diagnosis would allow clinicians to improve their care and would help policy makers in developing targeted guidelines. The aim of this cross-sectional study was thus to describe socio-demographic, clinical, and laboratory characteristics of TB/HIV co-infected patients at the time of TB diagnosis and treatment initiation in Yaoundé, Cameroon.

## Methods

### Study design and setting

The study was approved by Review Board of the Faculty of Medicine and Biomedical Sciences, University of Yaoundé 1. Written informed consent was obtained from the patient for the inclusion in this study. The retrospective, cross-sectional study focused on baseline characteristics of TB/HIV co-infected patients at the time when anti-tuberculous treatment was initiated. Data was collected from medical records in the Infectious Disease Unit (IDU) of the Yaoundé Central Hospital (YCH), Cameroon. The YCH houses an accredited treatment center, which is a major treatment center for HIV-infected patients in Cameroon. IDU of YCH manages 2% of all the TB cases in Cameroon annually [9]. All diagnosed TB cases are prescribed standard TB regimens and followed-up, with outcomes reported according to national TB control guidelines [9].

### Study population

TB cases were identified in the Tuberculosis Reporting Register of the IDU of the YCH, which is the official system for mandatory reporting of TB cases in the hospital. TB reporting is mandatory nationwide for new cases, relapses, and reentries after treatment dropout. All patients entered in the Register from January 1, 2006 to June 30, 2013 were evaluated for inclusion in the study. We included HIV-infected patients with TB, aged 15 years old or older.

### Data collection

We collected data from the TB registry of the IDU-YCH, from the hospitalization registry of the IDU, from medical records of hospitalized TB/HIV co-infected patients, and from the TB yellow cards of TB/HIV co-infected patients. A TB yellow card is a document on A4 format paper [9] used in anti-tuberculosis treatment. It contains the following information: the patient’s name, sex, age, addresses, TB localization, type of TB at treatment initiation, HIV status, and a calendar with check boxes for each day of taking anti-TB drugs.

We collected socio-demographic data including: age, sex, marital status, highest level of education, and residence. We also collected relevant clinical data. Patients were classified by the physician-in-charge of the CDT according to features of their clinical TB presentation, using the WHO standard international definitions [1] as one of the following: *smear-positive pulmonary TB* (Acid-fast bacilli [AFB] originally found in at least two sputum specimens)*, smear-negative pulmonary TB* (persistence of negative result of three sputum examinations after ten days of non-specific antibiotic treatment in a patient with clinical and radiographic signs suggesting pulmonary TB without other obvious cause)*, and extra-pulmonary TB* (a patient in whom TB was found in organs other than the lungs [e.g. pleura, lymph nodes, abdomen, genitourinary tract, skin, joints and bones, meninges]). *Extra-pulmonary TB* lies for the most part on the clinical decision based on suggestive clinical signs and evidence of a predominantly lymphocytic exudate as in the case of peritoneal TB with ascites or granulomatous lesions on histopathological examination of lymph nodes or other pieces of tissue obtained by biopsy. We defined as “*mixed form*” all presentations in which a patient had evidence of both pulmonary TB and extra-pulmonary TB. The WHO standard international definitions [1] were further used to categorize each person’s TB status at diagnosis: as either a *new case* or a *retreatment case*. Other clinical variables recorded from patients’ medical records included body weight; the presence of other AIDS-related opportunistic infections from clinical stages 2 to 4 as defined by WHO [10]; occurrence of non-TB and non-HIV related co-morbidities the most frequent being non-AIDS defining bacterial infections, malaria, diabetes, and hypertension; information on treatment outcomes; and whether or not the patient received Co-trimoxazole Prophylactic Therapy (CPT) and Anti-Retroviral Therapy (ART). Laboratory data included CD4 cell counts, hemoglobin level, and white blood cell counts.

### Data analysis

Data was coded, entered, and analysed using the Statistical Package for Social Science (SPSS) version 20.0 for Windows (IBM Corp. Released 2011. IBM SPSS Statistics for Windows, Version 20.0. Armonk, NY: IBM Corp.). We described continuous variables using means (standard deviation [SD]) or median (interquartile range [IQR]), and categorical variables using their frequencies (percentages). The chi-square test or Fisher’s exact test when appropriate, were used to compare categorical variables, Student’s t-test was used to compare means, and the Mann–Whitney U test was used to compare medians. All statistical tests were performed using two-sided tests at a 0.05 level of significance. Multiple imputation method was used to handle missing data, by creating a new data set, which was the average of five data sets of imputed values imputation [11,12]. This dataset has been used to compare body weight, CD4 count, hemoglobin level, and white blood cells count regarding sex and TB status at diagnosis.

## Results

**Figure 1:**
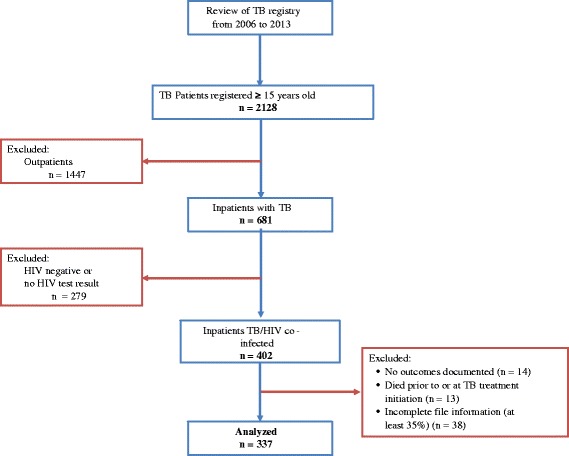
**Flowchart for TB/HIV inpatients in the infectious diseases unit of the Yaoundé Central Hospital (2006–2013).**

Figure 1 presents the flowchart of our study population. Of the 337 patient charts meeting inclusion criteria, we had missing values for weight in 36 records (10.7% of total), for CD4 counts in 28 records (8.3% of total), for white blood cell counts in 21 records (6.2% of total), and for hemoglobin levels in 21 records (6.2% of total).

### Socio-demographic, clinical, and laboratory profile of the study population

**Figure 2:**
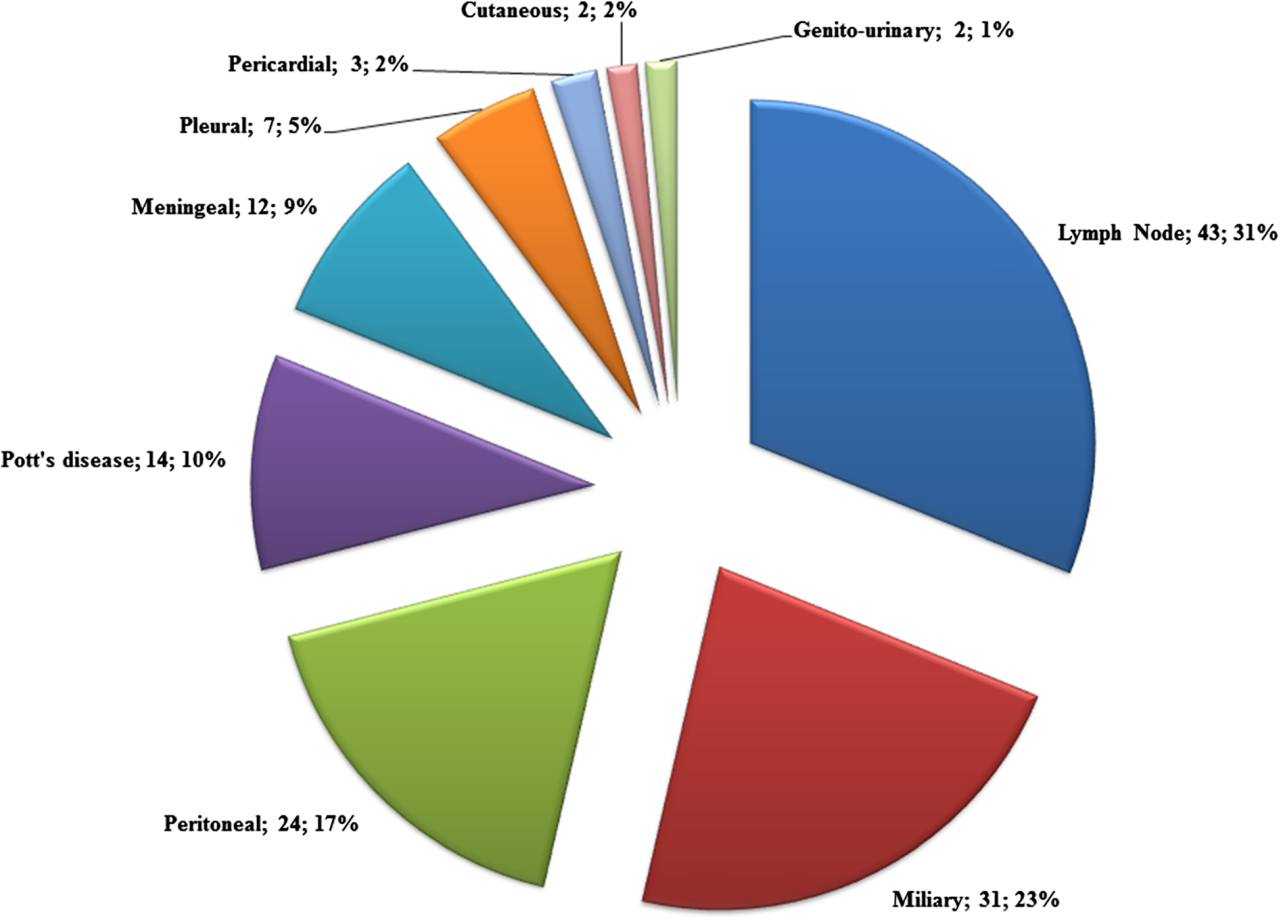
**Frequency of clinical forms of extra pulmonary TB among TB/HIV infected patients in Yaoundé Central Hospital from 2006 to 2013 (n = 138).** Data are: clinical form; n; proportion (%).

Table 1 presents our study cohort’s (n = 337) socio-demographic, clinical, and laboratory characteristics. The mean age of our patients was 39.3 years (SD 10.3) and the median was 38 years (IQR 32–46). There were slightly more women than men as 53.4% (n =180) were female. The greatest number had attained a secondary level of education (44.2%, n = 149). The majority of patients were married (46.0%, n = 155), closely followed by single status (44.8%, n = 151). The median duration of known HIV infection at the time of TB diagnosis was 0.97 (IQR 0.13-5.7) months and the mean was 8.2 (19.0) months. Patients’ mean body weight was 52.3 kg (SD 10.3). Most cases of tuberculosis represented new disease (88.1%, n = 297). Most of patients – over one third - had pulmonary TB alone (37.1%, n = 125), closely followed by extra-pulmonary disease (n = 119, 35.3%). Figure 2 details the distribution of the forms of extra-pulmonary tuberculosis in our patients. Lymph nodes (tuberculous lymphadenitis) were the most common extra-pulmonary site, representing a third of all extra-pulmonary tuberculosis (31.2%, n = 43) in our cohort. Other AIDS-related opportunistic infections and other (non-AIDS and non-TB) comorbidities were present in 53 (15.7%) and 40 (11.9%) patients respectively. Regarding HIV treatment, CPT and ART had been prescribed to 268 (79.5%) and 227 (67.4%) of the patients. The median CD4 count, white blood cells count, and hemoglobin level were 102 cell/mm^3^ (IQR 33–178), 5,100 cell/mm^3^ (3,300-7,990), and 8 g/dl (7–10) respectively.

**Table 1:** **Socio-demographic, clinical, and laboratory profiles of TB/HIV infected patients in Yaoundé Central Hospital (2006–2013), overall and by sex**

	**Male** **n = 157**	**Female** **n = 180**	***p***	**Total** **N = 337**
***Socio-demographic profile***				
Mean age, years	41.8 (9.2)	37.1 (10.7)	< .0001	39.3 (10.3)
Level of education				
• No formal	16 (10.2)	32 (17.8)	.042	48 (14.2)
• Primary	26 (16.6)	22 (12.2)		48 (14.2)
• Secondary	64 (40.8)	85 (47.2)		149 (44.2)
• University	51 (32.5)	41 (22.8)		92 (27.3)
Residence				
• Rural	22 (14.0)	14 (7.8)	.065	36 (10.7)
• Urban	135 (86.0)	166 (92.2)		301 (89.3)
Matrimonial status				
• Alone (single/divorced/widower)	59 (37.6)	123 (68.3)	< .0001	182 (54.0)
• Married	98 (62.4)	57 (31.7)		155 (46.0)
***Clinical profile***				
Clinical presentation of TB				
• PTB+ only	48 (30.6)	77 (42.8)	.020	125 (37.1)
• PTB- only	32 (20.4)	43 (23.9)		75 (22.3)
• EPTB only	65 (41.4)	54 (30.0)		119 (35.3)
• EPTB + PTB	12 (7.6)	6 (3.3)		18 (5.3)
TB status at diagnosis				
• New	135 (86.0)	162 (90.0)	.256	297 (88.1)
• Retreatment	22 (14.0)	18 (10.0)		40 (11.9)
Mean duration of known HIV infected, months	10.3 (22.1)	6.5 (15.8)	.076	8.2 (19.0)
Mean body weight, kg^¥^	55.4 (9.7)^α^	51.5 (10.4)^β^	.001	52.3 (10.3)
	55.6 (9.9)	51.3 (10.4)	.001	53.3 (10.4)
Presence of another (excluding TB) AIDS opportunist infections (stage 2 – 4 of WHO classification of HIV infection)	28 (17.8)	25 (13.96)	.321	53 (15.7)
Presence of other comorbidities, non-AIDS and non-TB	18 (11.5)	22 (12.2)	.830	40 (11.9)
Co-trimoxazole prophylactic therapy	125 (79.6)	143 (79.4)	.969	268 (79.5)
Antiretroviral therapy	112 (71.3)	115 (63.9)	.146	227 (67.4)
***Biological profile***				
Median CD4 count, cell/mm^3 ¥^	74 (27–193)^μ^	115 (42–172)^£^	.363	102 (33–178)
	75 (28–193)	116 (43–174)	.681	103 (34–181)
Median white blood cell count, cell/mm^3 ¥^	5,115 (3,300-7,800)^π^	5,100 (3,400-8,000)^ε^	.871	5,100 (3,300-7,990)
	5,100 (3250–7800)	5,200 (3,300-8,200)	.682	5,130 (3,300-8,000)
Median hemoglobin level, g/dl^¥^	9 (7–10)^π^	8 (6–10)^ε^	.003	8 (7–10)
	9 (7–10)	8 (6–10)	.002	8 (7–10)

Data are mean (standard deviation), n (%) and median (interquartile range).

Missing data: ^α^20, ^β^16, ^μ^10, ^£^18, ^π^7, and ^ε^11.

PTB+ and PTB-: smear –positive and –negative pulmonary TB.

EPTB: Extra pulmonary TB.

^¥^The row under this variable presents sensitivity analysis after multiple imputation of missing data.

### Comparison regarding sex and TB status at diagnosis

Table 1 includes a comparison of females and males in our study. Regarding socio-demographic characteristics, men were older than women (*p* < 0.001), more were married (*p* < 0.001) and had achieved a higher level of education (*p* = 0.042). As far as their clinical presentation, males had more smear-positive (PTB+) and smear negative (PTB-) pulmonary TB, less extra-pulmonary TB, and less mixed forms of TB (*p* = 0.020). Men also weighed more than women (*p* = 0.001). On laboratory evaluation, men had higher hemoglobin levels (*p* = 0.003). The patients with recurrent tuberculosis and classified as TB retreatment cases were older than patients with a new diagnosis of TB (*p* = 0.042) and had longer duration of known HIV infection (*p* = 0.004) (Table 2).

**Table 2:** **Comparison of socio-demographic, clinical, and laboratory profiles between patients with a new diagnosis of TB and those with retreatment TB in Yaoundé Central Hospital (2006–2013)**

	**Retreatment cases** **n = 40**	**New cases** **n = 297**	***p***
***Socio-demographic profile***			
Male	22 (55.0)	135 (45.5)	.256
Mean age, years	42.4 (8.6)	38.9 (10.4)	.042
Level of education			
• No formal	5 (12.5)	43 (14.5)	.956
• Primary	5 (12.5)	43 (14.5)	
• Secondary	18 (45.0)	131 (44.1)	
• University	12 (30.0)	80 (26.9)	
Residence			
• Rural	4 (10.0)	32 (10.8)	1.0
• Urban	36 (90.0)	265 (89.2)	
Matrimonial status			
• Alone (single/divorced/widower)	17 (42.5)	165 (55.6)	.120
• Married	23 (57.5)	132 (44.4)	
***Clinical profile***			
Clinical presentation of TB			
• PTB+ only	16 (40.0)	109 (36.7)	.975
• PTB- only	8 (20.0)	65 (22.6)	
• EPTB only	14 (35.0)	105 (35.4)	
• EPTB + PTB	2 (5.0)	16 (5.4)	
Mean duration of known HIV infected, months	22.9 (33.6)	6.3 (15.2)	.004
Mean body weight, kg^¥^	53.2 (9.5)^α^	53.2 (10.4)^β^	.996
	53.2 (9.4)	53.3 (10.5)	.941
Presence of another (excluding TB) AIDS opportunist infections (stage 2 – 4 of WHO classification of HIV infection)	4 (10.0)	49 (16.5)	.286
Presence of another comorbidities, no AIDS-no TB	8 (20.0)	32 (10.8)	.114
Co-trimoxazole prophylactic therapy	32 (80.0)	236 (79.8)	.937
Antiretroviral therapy	32 (80.0)	195 (65.7)	.069
***Biological profile***			
Median CD4 count, cell/mm^3 ¥^	74 (19–169)	102 (34–181)^£^	.298
	74 (19–169)	105 (35–184)	.238
Median white blood cell count, cell/mm^3 ¥^	5,750 (3,825-8,045)	5,000 (3,290-7,900)^ε^	.615
	5,750 (3,825-8,045)	5,100 (3,260-8,000)	.538
Median hemoglobin level, g/dl^¥^	9 (7–10)	8 (7–10)^ε^	.846
	9 (7–10)	8 (7–10)	.802

Data are mean (standard deviation), n (%) and median (interquartile range).

Missing data:^α^2, ^β^34, ^£^28, and ^ε^18.

PTB+ and PTB-: smear -positive and –negative pulmonary TB.

EPTB: Extra pulmonary TB.

^¥^The row under this variable presents sensitivity analysis after multiple imputation of missing data.

## Discussion

The most frequent clinical presentation of patients diagnosed with tuberculosis in the setting of HIV co-infection was that of smear-positive pulmonary TB, just as has been observed from reports of TB and HIV co-infection from other geographic areas outside of sub-Saharan Africa [13]. The majority of our cases represented newly diagnosed TB and presented in an advanced state of immune deficiency, with low CD4 T-cell counts. We found low frequencies of AIDS-related opportunistic infections and of other (non-AIDS and non-TB) comorbidities. The extent of CPT and ART in our study is consistent with data recently reviewed and reported by the Centers for Disease Control [2] and fails to achieve the recommended treatment goals of the WHO and the Cameroon’s Ministry of Public Health [9].

### Socio-demographic profile

Currently, there is no conclusive evidence of sex-based differences in the occurrence of TB and HIV co-infection in developing countries [6,14-17]. Our study also had a slight preponderance of co-infected women (53.4%) as compared to men. The apparent sex-based difference is probably related to the high incidence of HIV infection among females in sub-Saharan Africa in general [17,18] and Cameroon in particular [5,18]. Women, who have a higher susceptibility to HIV infection [19-21], are usually exposed to sexual activity earlier than men in francophone sub-Saharan Africa, and particularly in Cameroon [22].

In terms of age distribution, the median age was 38 years (IQR 32–46 years) and the mean was 39.3 (SD10.3 years). This is consistent with the findings of other studies [14,23-27]. The TB/HIV co-infection age distribution likely reflects the age-specific prevalence of HIV in the community. This in turn relates to patients’ being in a sexually active age group and encountering sexual partners in whom both TB and HIV are prevalent. In our study, the men with TB and HIV were significantly older than the women. In Cameroon, HIV prevalence is higher in young females compared to young males [5]. Furthermore, the age group most affected by HIV infection in women is 35–39 years (HIV prevalence 10.0%) and in men is 45–49 years (HIV prevalence 6.3%) [5]. Age was also greater among the patients undergoing retreatment of TB as compared to new TB cases. This result is not surprising and is logical, as the passage of time and ageing increase the risk of TB relapse.

In our population, a secondary level of education was the most representative with 44.2% of patients, although men completed a higher level of education than did women. Our results are consistent with others studies [5,16,27]. Findings from a nationwide survey in Cameroon [5] revealed that HIV prevalence is highest among those with primary or secondary/1^st^ cycle education; the same would be thus expected for those with HIV complicated by the emergence of TB.

In the present study, the majority of patients (89.3%) resided in an urban area. Studies, particularly those in sub-Saharan African countries, have shown that the number TB/HIV co-infected cases is higher in urban than rural areas [8]. It is also important to note however, that our study was conducted in a tertiary-care hospital located in an urban area. The Yaoundé area’s inhabitants are typical of the urban population of Cameroon.

Almost half of the patients were married (46.0%). In our study, more men than women were married. There are several possible explanations. A man may be more likely to abandon his wife when she becomes ill (especially in case of HIV infection) and then remarry; also, polygamy is authorized in Cameroon, so if a man is abandoned by one of his wives, he may have others wives and thus stay married. Finally, because of cultural, economic, societal, and religious constraints, women are overall more likely to remain in a marriage than men.

### Clinical profile

In our study, the most frequent clinical presentation of tuberculosis was smear-positive pulmonary disease alone (37.1%), followed by extra-pulmonary TB (35.3%). There have been conflicting reports on the frequency of the various clinical presentations of TB in TB/HIV co-infected patients. Several studies have shown a higher occurrence of smear-negative pulmonary TB [16,26,28,29], while others have shown a high occurrence of extra-pulmonary TB [30]. Our findings are similar to those of Pefura et *al.* in Yaoundé, Cameroon [8] and to other studies from Malaysia and Brazil [23,24]. Those reports differ from other published data reporting a greater proportion of smear-negative pulmonary TB and extra-pulmonary TB diagnoses in TB/HIV co-infected patients, particularly in a background of advanced immune deficiency (AIDS) where TB tends to present atypically and to more frequently disseminate outside the lung [31]. We acknowledge that one explanation for our results - obtained from a hospital inpatient setting - could be the fact that patients with smear-positive pulmonary TB are more likely than smear negative patients to be hospitalized in order to prevent transmission of the bacilli to others.

Of the 121 (35.9%) patients with isolated extra-pulmonary TB and 16 (4.7%) with the mixed clinical form, tubercular lymphadenitis was the most frequent presentation (31.2%). Our findings are in agreement with others in developing countries [30,32-34]. Indeed, this reflects the profound host immune suppression induced by HIV infection, which facilitates the dissemination of *Mycobacterium tuberculosis* out of the lungs, via lymphangitic spread [35] and causes the reactivation of infection in extra-pulmonary organs [36]. However, the relative diagnostic ease with which lymph node TB is detected and diagnosed as compared to other forms of extra-pulmonary TB in resource-limited settings such as Cameroon, may be considered as a partial explanation for the higher percentage of lymph node TB.

The median duration of known HIV infection when anti-tuberculous treatment was initiated was 0.97 (IQR 0.13-5.7) months. Apart from TB, 15.7% of patients had an additional AIDS-related opportunistic disease and 11.9% had other co-morbid conditions, which were neither TB nor AIDS-related opportunistic diseases or infections. These findings are congruent with those in Malaysia [23] and likely reflect the fact that the occurrence of tuberculosis is the first manifestation of AIDS in most patients.

The median weight of our patients was 53 kg. Very few studies have looked at this variable and our study shows similar findings to the published literature [23,26]. Unfortunately, we could not obtain data as body mass index. Weight loss is common in the setting of both TB and HIV infection [37]. In our study, the men’s body mass was greater than that of women, which is expected.

At TB treatment initiation, the majority of patients (79.5%) were receiving CPT. This is higher than the coverage observed in several studies [16,25,38]. In Cameroon, CPT is indicated in all cases of TB/HIV co-infection both in adults and children, irrespective of the CD4 cell count level and WHO clinical stage [9]. This implies all the patients in our cohort were eligible for CPT but the complete coverage (100%) was not attained. The mindset of some clinicians who place more emphasis on prescribing ART, regarded as lifesaving, while unfortunately neglecting CPT might be an explanation for this finding. It may also be possible that busy CDT staff neglected to consistently document adherence to CPT prescribing in the medical record. The occurrence of insufficient medication supplies and the inadequacy of CPT stocks at the CDT requiring patients to obtain medication elsewhere on their own are also possible explanations.

More than half of the patients (67.4%) had started ART, yet the frequency of ART coverage among TB/HIV co-infected patients remained less than the 80% coverage target set by the Ministry of Public Health in Cameroon [9]. Despite recommendations of WHO (all HIV-infected patients must be on ART), some clinicians remain reluctant to prescribe ART to their HIV-infected TB patients, due to concerns about overlapping toxicities, pill burden, and immune reconstitution inflammatory syndrome [39]. Nonetheless, the extent of ART coverage for TB/HIV co-infected patients in studies carried out in North-West region in Cameroon [38], in Southwest Ethiopia [26], and in Trinidad and Tobago [25] was less than what we found. One important factor underlying the difference in the extent of coverage is the fact that all our patients were hospitalized, and so, it was easier to administer ART to patients and to and ensure follow-up.

### Laboratory profile

In an immunocompetent host, infection causes elevation of white blood cell counts. In TB/HIV co-infected patients however, as infection progresses, there is depletion of neutrophils and monocytes by *Mycobacterium tuberculosis* and lymphocytes (CD4 especially) by HIV [40]. In this study, total white blood cell counts were documented prior to TB treatment initiation; the median white blood cell count was 5,100 cells/mm^3^ (normal range). A study carried out in Malaysia by Ismail et *al.* reports similar findings [23].

The median hemoglobin level in our patients was 8 g/dl, comparable to what Ismail et *al.* found in their population [23]. In TB patients, anemia may occur as a result of suppression of erythropoiesis by inflammatory mediators [40]. Anemia in HIV-positive patients has an extensive differential diagnosis, which includes lympho-proliferative disorders, abnormal iron metabolism, hemolysis, and bone marrow suppression (especially with zidovudine) [41,42]. Our study found a difference between males and females regarding hemoglobin concentration, as expected. It is well established that the hemoglobin concentration is physiologically lower in women [43].

Our study shows that, at the time of inpatient TB treatment initiation, most patients were at an advanced immune deficiency state as the median CD4 T-cell count was 102 cells/mm^3^. This finding is similar to other studies in developing countries [16,23,25,27,30]. HIV impairs the host’s immune system [44], setting the stage for the emergence of opportunistic infections like TB, as in our study subjects, and further depletion of CD4 cells [45] ultimately leading to illness requiring medical intervention. Altogether, the synergism between HIV and TB is a reflected in the level of CD4 cell counts measured in our patients [45,46].

### Limitations

Our study captured patients undergoing treatment for tuberculosis over a 90-month period, from January 1, 2006 through June 30, 2013. Starting in 2012, guidelines issued by the Cameroonian Ministry of Public Health for prescribing ART changed. Prior to 2012, it was recommended that ART therapy in the setting of HIV be started when the CD4 cell count was less than 200 cells/mm^3^; the 2012 revision instead advised starting ART once the CD4 cell count dropped to 350 cells/mm^3^. This likely lead to more of our patients being started on ART later in the study period; tuberculosis guidelines however, remained unchanged for the entire period of our study. Some data for body weight, hemoglobin level, white blood cell count, and CD4 count were missing. In those four categories, the range of missing data was between 6.2 and 10.7%. The reasons for the missing data included failure to enter information in the medical treatment records, and the inability for some patients to pay for laboratory evaluations, which were thus not obtained. Whatever the underlying cause, the missing data underscore the challenges of conducting “real-life” clinical research in resource-limited settings, like Cameroon and other sub-Saharan countries. Aggregate data derived from laboratory testing should be interpreted cautiously as technological advances in the measurement of hematological and immunological parameters over the 8 years of our study, with concurrent changes in methodologies and in laboratory equipment preclude making precise comparisons over time. In addition, as some patients had their laboratory examinations performed in settings other than the YCH, including in private laboratories, we could not retrospectively ascertain which autoanalyzers and laboratory equipment were used to perform those exams.

## Conclusions

In a population of hospitalized Cameroonian patients beginning TB treatment in the setting of HIV co-infection, we found that most of the patients were married, lived in urban areas, and had attained a secondary level of education. Women were younger and fewer were married, as compared to the men. The most common TB presentation was smear-positive pulmonary TB, closely followed by extra-pulmonary TB. Most of our TB cases were new cases; there were few other AIDS-related opportunistic diseases and few non-AIDS related comorbidities. Most patients were at an advanced stage of immunosuppression. Adherence to the WHO and the Cameroonian Ministry of Public Health recommendations of TB and HIV co-infection was suboptimal and needs to be improved. In the light of this study, we urge clinicians to prescribe ART for all persons co-infected with TB and HIV and to also treat all HIV infected individuals with CPT, as per the most recently issued treatment guidelines. We further recommend that the Ministry of Health continue promoting and implementing guidelines for the best management of HIV-infected patients and especially for those with TB complicating HIV, by targeting all health care personnel in a concerted educational outreach.

## Abbreviations

AIDS: Acquired immune deficiency syndrome
ART: Anti-retroviral therapy
CD4: Cluster of differentiation 4
CDC: Centers for disease control
CDT: Center of diagnosis and therapy
CPT: Co-trimoxazole prophylactic therapy
HIV: Human immunodeficiency virus
IDU: Infectious diseases unit
TB: Tuberculosis
WHO: World Health Organization
YCH: Yaoundé Central Hospital
